# A Transcriptome Array-Based Approach to Link SGLT-2 and Intrarenal Complement C5 Synthesis in Diabetic Nephropathy

**DOI:** 10.3390/ijms242317066

**Published:** 2023-12-02

**Authors:** Peter Korsten, Björn Tampe

**Affiliations:** Department of Nephrology and Rheumatology, University Medical Center Göttingen, 37075 Göttingen, Germany; peter.korsten@med.uni-goettingen.de

**Keywords:** diabetic nephropathy, immunology, SGLT-2 inhibitor, innate immunity, complement synthesis, metabolic dysregulation

## Abstract

Diabetic nephropathy is a common microvascular complication of diabetes mellitus. It is characterized by progressive chronic kidney disease (CKD) with decline of kidney function by hyperfiltration. On a mechanistic level, activation of the complement system has been implicated in the pathogenesis of diabetic nephropathy. Therefore, here we pursued a transcriptome array-based approach to link intrarenal SGLT-2 and the synthesis of distinct complement components in diabetic nephropathy. Publicly available datasets for *SLC5A2* (encoding SGLT-2) and complement system components were extracted specifically from microdissected tubulointerstitial (healthy controls: *n* = 31, diabetic nephropathy: *n* = 17) and glomerular compartments (healthy controls: *n* = 21, diabetic nephropathy: *n* = 12). First, we compared tubulointerstitial and glomerular log_2_ *SLC5A2* mRNA expression levels and confirmed a predominant synthesis within the tubulointerstitial compartment. Among various complement components and receptors, the only significant finding was a positive association between *SLC5A2* and the tubulointerstitial synthesis of the complement component C5 in diabetic nephropathy (*p* = 0.0109). Finally, intrarenal expression of *SLC5A2* was associated predominantly with pathways involved in metabolic processes. Interestingly, intrarenal complement C5 synthesis was also associated with enrichment of metabolic signaling pathways, overlapping with *SLC5A2* for “metabolism” and “biological oxidations”. These observations could be of relevance in the pathogenesis of diabetic nephropathy and implicate a mechanistic link between SGLT-2 and intrarenal complement synthesis.

## 1. Introduction

Diabetic nephropathy is a common microvascular complication of diabetes mellitus. It is characterized by progressive chronic kidney disease (CKD) with the decline of kidney function by hyperfiltration. Diabetic nephropathy is the leading cause of chronic and end-stage kidney disease (ESKD) worldwide [1,2]. Importantly, diabetic nephropathy is associated with increased cardiovascular morbidity and mortality [2]. For treatment of diabetic nephropathy to slow down CKD progression, angiotensin-converting enzyme inhibitors (ACEi), angiotensin II receptor antagonists (ARB), and the sodium-glucose cotransporter 2 inhibitors (SGLT-2is) are approved. On a mechanistic level, activation of the complement system has been implicated in the pathogenesis of diabetic nephropathy. Proteomic analysis in human kidney biopsies showed increased complement deposition in patients with diabetic nephropathy [3]. Hyperglycemia has been shown to activate the lectin pathway by glycation of pattern recognition molecules and complement regulatory proteins, resulting in intrarenal activation of the complement system and tubulointerstitial injury in diabetic nephropathy [4,5]. Compared to other interventions, no prior trial of any therapeutic agent in diabetic nephropathy has demonstrated an effect of the magnitude observed with SGLT-2i treatment [6]. Because complement is increasingly recognized in the pathogenesis of diabetic nephropathy, SGLT-2is may also exert nephroprotection by affecting complement system activation and signaling in diabetic nephropathy to achieve such beneficial effects [1].

In general, systemic or locally produced complement activation has also been implicated in the disease pathogenesis of antibody-mediated glomerular diseases [1]. We have already described that various autoimmune diseases are characterized by intrarenal complement depositions, at least in part driven by local synthesis of complement components, serving as biomarkers and direct mediators of injury [7,8,9,10,11]. Due to complement activity in many tissues, the consequences of its dysregulation are extensive, and increasing evidence has suggested the potential clinical relevance of complement pathway intervention. In diabetic nephropathy, systemic consumption of complement components and associated hypocomplementemia have not been described, suggesting a local synthesis of complement components [12]. Therefore, here we pursued a transcriptome array-based approach to link intrarenal SGLT-2 and the synthesis of distinct complement components in diabetic nephropathy. Particularly, we have included a dataset on diabetic nephropathy where a genome-wide transcriptome analysis already shows upregulation of the complement pathway [13].

## 2. Results

### 2.1. Predominant Tubulointerstitial SLC5A2 Expression in Healthy Controls and Diabetic Nephropathy

**Figure 1:**
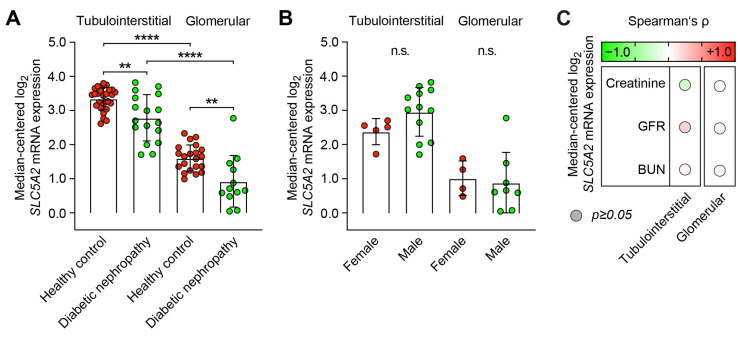
Predominant tubulointerstitial *SLC5A2* expression in healthy controls and diabetic nephropathy. (**A**) Median-centered log_2_ *SLC5A2* expression levels in microdissected tubulointerstitial (healthy controls: *n* = 31, diabetic nephropathy: *n* = 17) and glomerular compartments (healthy controls: *n* = 21, diabetic nephropathy: *n* = 12) are shown by scatter dot plots including mean ± SD. Comparisons of groups were performed using the Mann–Whitney U-test to determine differences in medians (** *p <* 0.01, **** *p* < 0.0001). (**B**) Median-centered log_2_ *SLC5A2* mRNA expression levels in diabetic nephropathy are shown by scatter dot plots including mean ± SD with group separation for female and male sex. Comparisons of groups were performed using the Mann–Whitney U-test to determine differences in medians (n.s: not significant). (**C**) Correlations between tubulointerstitial and glomerular *SLC5A2* mRNA expression levels and laboratory markers of kidney function in diabetic nephropathy are shown by heatmap reflecting mean values of Spearman’s *p*; circle size represents significance level.

First, we compared tubulointerstitial and glomerular log_2_ *SLC5A2* mRNA expression levels and confirmed a predominant synthesis within the tubulointerstitial compartment in healthy controls and diabetic nephropathy (Figure 1A and Tables S1 and S2) [14]. Interestingly, we identified decreased *SLC5A2* mRNA expression in diabetic nephropathy as compared to healthy controls (Figure 1A). Specifically, in diabetic nephropathy, there were no differences between female and male patients (Figure 1B). Furthermore, there was an association with kidney function impairment as reflected by serum creatinine, glomerular filtration rate (GFR), or blood urea nitrogen (BUN, Figure 1C).

### 2.2. Intrarenal SLC5A2 Expression Is Associated with Tubulointerstitial Synthesis of Distinct Complement Components

**Figure 2:**
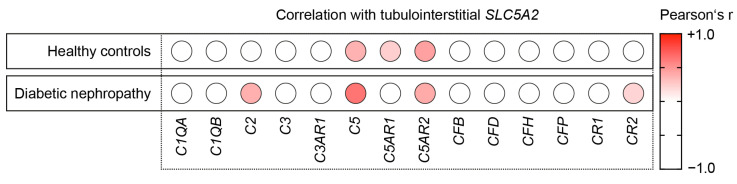
Intrarenal *SLC5A2* expression is associated with tubulointerstitial synthesis of distinct complement components. Correlations between tubulointerstitial *SLC5A2* mRNA expression levels and various complement components in healthy controls (*n* = 31) and diabetic nephropathy (*n* = 17) are shown by heatmap reflecting mean values of Spearman’s *p*; circle size represents significance level.

Because we found a predominant tubulointerstitial expression of *SLC5A2* in both healthy controls and diabetic nephropathy, we next analyzed the association between tubulointerstitial *SLC5A2* mRNA expression levels, focusing on various complement components and receptors. Among them, there were positive correlations with complement components *C5* and receptors *C5AR1* and *C5AR2* in healthy controls (Spearman’s *p* > 0.2, Figure 2). In diabetic nephropathy, we identified positive correlations specifically with complement components *C2* and *C5* and receptors *C5AR2* and *CR2* (Figure 2).

### 2.3. Intrarenal SLC5A2 Expression Is Associated with Tubulointerstitial Synthesis of Complement C5 in Diabetic Nephropathy

We next analyzed the relevance of tubulointerstitial *SLC5A2* mRNA expression in association with the identified synthesis of distinct complement components, specifically in diabetic nephropathy. As assessed by stepwise logistic regression, the only significant finding was a positive association between *SLC5A2* and the tubulointerstitial synthesis of the complement component *C5* (*p =* 0.0109, Table 1). 

### 2.4. Intrarenal SLC5A2 Expression and Complement C5 Synthesis Associated with Distinct Molecular Signatures in Diabetic Nephropathy

**Figure 3:**
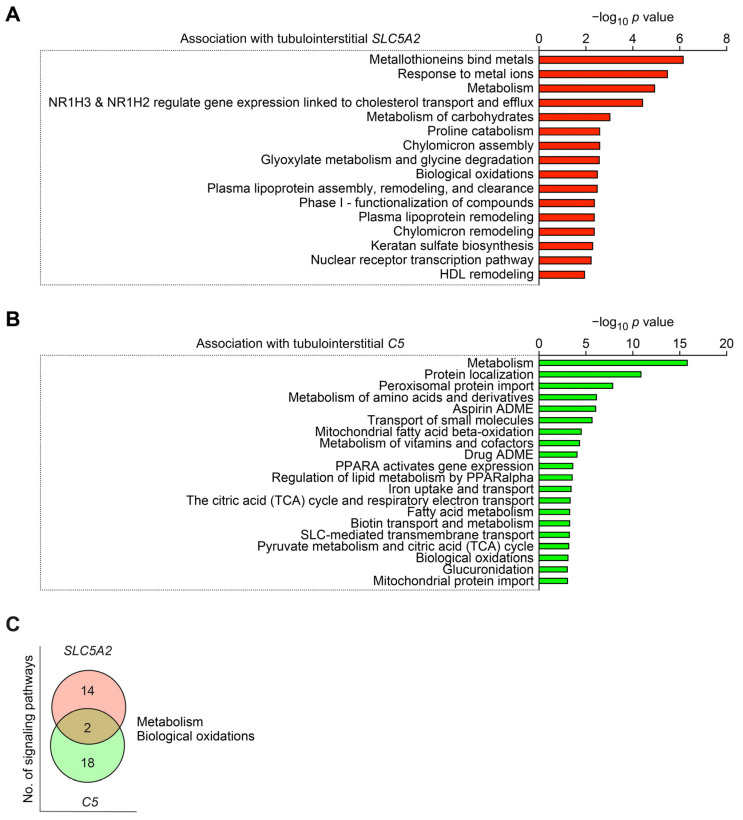
Intrarenal *SLC5A2* expression and complement *C5* synthesis associated with distinct molecular signatures in diabetic nephropathy. (**A**) Entities −log_10_ *p* values of signaling pathways associated with tubulointerstitial *SLC5A2* mRNA expression in diabetic nephropathy are shown. (**B**) Entities −log_10_ *p* values of signaling pathways associated with tubulointerstitial *C5* mRNA expression in diabetic nephropathy are shown. (**C**) Number of signaling pathways associated with either tubulointerstitial *SLC5A2*, complement *C5* mRNA expression, or both in diabetic nephropathy are shown.

Finally, we aimed to identify molecular signatures associated with *SLC5A2* and intrarenal synthesis of complement component *C5* [13]. Intrarenal expression of *SLC5A2* was associated predominantly with pathways involved in metabolic processes (Figure 3A and Table S3). Interestingly, intrarenal complement *C5* synthesis was also associated with enrichment of metabolic signaling pathways (Figure 3B and Table S4), overlapping with *SLC5A2* for “metabolism” and “biological oxidations” (Figure 3C). In summary, we confirm a predominant tubulointerstitial expression of SGLT-2 in diabetic nephropathy. Furthermore, tubulointerstitial SGLT-2 correlated explicitly with the tubulointerstitial synthesis of complement component C5 in diabetic nephropathy.

## 3. Discussion

SGLT-2i exerts unequivocal nephroprotective effects by reducing glomerular hyperfiltration and albuminuria, tubular injury, loss of kidney function, and incidence of acute kidney injury. Although the mechanisms by which SGLT-2i can protect kidney function are not fully understood, it has previously been proposed that other pathways, including suppression of inflammation and fibrosis, are involved [15,16]. This is also relevant to autoimmune kidney diseases, including ANCA-associated renal vasculitis and lupus nephritis [17,18,19]. Interestingly, it has recently been reported that SGLT-2i exerts nephroprotection by attenuating autoimmunity, at least in part attributed to complement system modulation [20]. In the present study, we expand our current knowledge and report novel findings linking SGLT-2 and complement synthesis in diabetic nephropathy. Particularly, this comparative analysis revealed a predominant tubulointerstitial expression of SGLT-2 in both healthy controls and diabetic nephropathy. This is in line with previous observations confirming the abundance of SGLT-2 predominantly in the proximal tubular epithelium but also in glomerular podocytes [18,21,22]. Furthermore, we identified a positive correlation between SGLT-2 and tubulointerstitial synthesis of distinct complement components. Specifically in diabetic nephropathy, we here identified a positive association between SGLT-2 expression and the tubulointerstitial synthesis of the complement component C5. Evidence of glycated complement components was first described in patients with diabetic kidney disease more than three decades ago [23]. In this context, glycated complement components C3 and C4 are observed in the early stages of diabetic nephropathy [24]. Regarding complement component C5, tubular depositions have already been shown to correlate with the severity of diabetic nephropathy in humans [25]. Moreover, there is evidence for a pivotal role of the complement component C5 and its anaphylatoxin C5a signaling axis in contributing to kidney injury in experimental diabetic nephropathy [26,27]. Complement C5a and its receptor C5aR1 are upregulated early in the disease process and prior to manifesting kidney injury in several experimental models of diabetic nephropathy [26]. Particularly, C5aR1 has been shown to be expressed on proximal tubular cells, podocytes, fibroblasts, mesangial cells, and vascular endothelial and smooth muscle cells [28,29,30,31]. Finally, blocking C5a/C5a receptor 1 signaling has been shown to ameliorate interstitial fibrosis in experimental diabetic nephropathy [26]. Regarding molecular mechanisms, we here identified that intrarenal complement C5 synthesis was associated with the enrichment of metabolic signaling pathways, overlapping with SGLT-2 for “metabolism” and “biological oxidations”. This is in line with previous findings in experimental diabetic nephropathy confirming that C5 signaling is predominantly involved in immunometabolic signaling pathways [26]. Furthermore, several signaling pathways have already been implicated in linking increased oxidative stress and activation of inflammatory pathways to progressive kidney function decline in diabetic nephropathy [32,33]. This could be of relevance in the pathogenesis of diabetic nephropathy and could implicate a mechanistic link between SGLT-2 and intrarenal complement synthesis. Because the dataset included in our study was generated from an ethnically diverse population with various histopathological lesions, our findings could be of relevance for a large patient population, as it has been observed for nephroprotection by SGLT-2is in diabetic nephropathy [13,14,34].

Since new therapeutics targeting the complement system are emerging, these agents could be potentially beneficial in diabetic nephropathy. The recombinant humanized monoclonal antibody eculizumab is selective against C5 and inhibits its cleavage, reducing the release of anaphylatoxin C5a. Beneficial effects, such as temporary stabilization of renal function or reduction in albuminuria, have already been observed with eculizumab in progressive IgA nephropathy [35]. The first long-acting complement inhibitor, ravulizumab, has similar effects by C5 antagonism and is currently under preclinical evaluation in IgA nephropathy [36]. Additionally, a randomized phase II study is currently evaluating the efficacy and safety of ravulizumab in IgA nephropathy patients (NCT04564339). Furthermore, cemdisiram (ALN-CC5) as synthetic RNAi that blocks hepatic C5 production is also being tested in a phase II study in patients at high risk of progressive IgA nephropathy (NCT03841448). Regarding C5a receptor 1 (C5aR1) inhibition, avacopan (CCX168) is an orally active small molecule that selectively antagonizes C5aR1. Avacopan is currently approved for the treatment of ANCA-associated systemic vasculitis and evaluated for the treatment of IgA nephropathy (NCT02384317) [37]. Although we are aware that these observations require further validation with regard to protein expression and experimental models, here we link SGLT-2 and intrarenal synthesis of complement C5 in diabetic nephropathy, as it has also been recently described in experimental lupus nephritis [20].

## 4. Materials and Methods

### 4.1. Analyses of Publicly Available Array Datasets

Datasets provided publicly were analyzed according to general recommendations [38,39]. Publicly available datasets for *SLC5A2* (encoding SGLT-2) and complement system components of mRNA expression were extracted from Nephroseq (www.nephroseq.org, accessed on 8 April 2023, University of Michigan, Ann Arbor, MI, USA). Particularly, median-centered log_2_ *SLC5A2* mRNA expression levels (reporter ID: 6524, platform: Affymetrix Human Genome U133 Plus 2.0 Array, altCDF v10), *C1QA* (reporter ID: 712), *C1QB* (713), *C2* (717), *C3* (718), *C3AR1* (719), *C5* (727), *C5AR1* (728), *GPR77* (encoding C5AR2, 27202), *CFB* (629), *CFD* (1675), *CFH* (3075), *CFP* (5199), *CR1* (1378), and *CR2* (1380) were extracted specifically from microdissected tubulointerstitial (healthy controls: *n* = 31, diabetic nephropathy: *n* = 17) and glomerular compartments (healthy controls: *n* = 21, diabetic nephropathy: *n* = 12, Tables S1 and S2) [14]. From the same dataset, patient demographics and laboratory parameters for kidney function were also available and included in the analysis [14]. For multivariate analysis, mean values of Spearman’s ρ > 0.2 were included. Pathway analysis was performed separately for gene enrichment associated with tubulointerstitial *SLC5A2* and *C5* mRNA expression (healthy controls: *n* = 12, diabetic nephropathy: *n* = 10) [13]. Associated genes with a correlation threshold of ≥0.5 were included for pathway analysis by using reactome (http://reactome.org, accessed on 20 April 2023), and pathways with a predefined entity value of *p ≤* 0.001 were included and shown in Tables S3 and S4 [40].

### 4.2. Statistical Methods

Spearman’s correlation was used to assess the correlation between continuous variables; multivariate analysis was performed by stepwise linear regression presenting beta coefficients (*ß*) and values of *p*. Data analyses were performed using GraphPad Prism (version 9.3.1 for macOS, GraphPad Software, San Diego, CA, USA) and IBM SPSS Statistics (version 29.0.0.0 for MacOS, IBM Corporation, Armonk, NY, USA); a value of *p <* 0.05 was considered statistically significant.

## 5. Conclusions

Here, we show a positive association between *SLC5A2* and the tubulointerstitial synthesis of the complement component *C5*. Intrarenal expression of *SLC5A2* was associated predominantly with pathways involved in metabolic processes. These observations could be of relevance in the pathogenesis of diabetic nephropathy and implicate a mechanistic link between SGLT-2 and intrarenal complement synthesis.

## Figures and Tables

**Table 1:** Multiple comparisons between *SLC5A2* and the tubulointerstitial synthesis of the complement components in diabetic nephropathy.

Variable	*ß*	*p* Value
*C2*—median-centered log_2_ mRNA expression	0.2981	0.1633
*C5*—median-centered log_2_ mRNA expression	0.5997	0.0109
*C5AR2*—median-centered log_2_ mRNA expression	0.3918	0.0572
*CR2*—median-centered log_2_ mRNA expression	0.0976	0.6626

## Data Availability

The original contributions presented in the study are included in the article/Supplementary Materials; further data and material are available from the corresponding author upon reasonable request.

## Supplementary Materials

The supporting information can be downloaded at: https://www.mdpi.com/article/10.3390/ijms242317066/s1.
