# Supplementary material for: A Transcriptome Array-Based Approach to Link SGLT-2 and Intrarenal Complement C5 Synthesis in Diabetic Nephropathy
Source: Int J Mol Sci. 2023 Dec 2;24(23):17066. doi: 10.3390/ijms242317066 (PMC10707485; doi:10.3390/ijms242317066)
Supplement: Supplementary file 1 [file ijms-24-17066-s001.zip › ijms-2640768-supplementary.pdf]

A transcriptome array-based approach to link SGLT-2 and intrarenal complement C5 synthesis in diabetic nephropathy

Supplemental Material

Peter Korsten[1], Björn Tampe[1,*]

[1]Department of Nephrology and Rheumatology, University Medical Center Göttingen, Göttingen, Germany

**Corresponding author*

Corresponding author:

Björn Tampe, MD

Department of Nephrology and Rheumatology

University Medical Center Göttingen, Germany

Telephone number: +49 551 39 10575

Email: bjoern.tampe@med.uni-goettingen.de

**Table S1. Included samples extracted from Nephroseq for tubulointerstitial *SLC5A2* mRNA expression in diabetic nephropathy.**			
**Sample name**	**Patient ID**	**Group**	**$\log_2$ *SLC5A2***
H1Tub.HGU133A.DNNULL.DN1	DN1	Diabetic Nephropathy	3,09435
H1Tub.HGU133A.DNNULL.DN10	DN10	Diabetic Nephropathy	2,63963
H1Tub.HGU133A.DNNULL.DN11	DN11	Diabetic Nephropathy	3,81952
H1Tub.HGU133A.DNNULL.DN2	DN2	Diabetic Nephropathy	2,70262
H1Tub.HGU133A.DNNULL.DN3	DN3	Diabetic Nephropathy	1,7074
H1Tub.HGU133A.DNNULL.DN5	DN5	Diabetic Nephropathy	2,50726
H1Tub.HGU133A.DNNULL.DN6	DN6	Diabetic Nephropathy	2,55282
H1Tub.HGU133A.DNNULL.DN7	DN7	Diabetic Nephropathy	3,02197
H1Tub.HGU133A.DNNULL.DN8	DN8	Diabetic Nephropathy	3,49134
H1Tub.HGU133A.DNNULL.DN9	DN9	Diabetic Nephropathy	1,99601
H7Tub.HGU133Plus2.DN1101.DN1101	DN1101	Diabetic Nephropathy	3,70394
H7Tub.HGU133Plus2.DN1108.DN1108	DN1108	Diabetic Nephropathy	2,05049
H7Tub.HGU133Plus2.DN1110.DN1110	DN1110	Diabetic Nephropathy	1,72055
H7Tub.HGU133Plus2.DN1114.DN1114	DN1114	Diabetic Nephropathy	3,37274
H7Tub.HGU133Plus2.DN1132.DN1132	DN1132	Diabetic Nephropathy	3,59363
H7Tub.HGU133Plus2.DN1133.DN1133	DN1133	Diabetic Nephropathy	2,99347
H7Tub.HGU133Plus2.DN1139.DN1139	DN1139	Diabetic Nephropathy	2,41642

**Table S2. Included samples extracted from Nephroseq for glomerular *SLC5A2* mRNA expression in diabetic nephropathy.**			
**Sample name**	**Patient ID**	**Group**	**$\log_2$ *SLC5A2***
H1Glom.HGU133A.DNNULL.DN1	DN1	Diabetic Nephropathy	0,60626
H1Glom.HGU133A.DNNULL.DN10	DN10	Diabetic Nephropathy	0,65666
H1Glom.HGU133A.DNNULL.DN2	DN2	Diabetic Nephropathy	0,48519
H1Glom.HGU133A.DNNULL.DN5	DN5	Diabetic Nephropathy	1,26904
H1Glom.HGU133A.DNNULL.DN8	DN8	Diabetic Nephropathy	1,45295
H7Glom.HGU133Plus2.DN901.DN901	DN901	Diabetic Nephropathy	0,5905
H7Glom.HGU133Plus2.DN910.DN910	DN910	Diabetic Nephropathy	0,87945
H7Glom.HGU133Plus2.DN914.DN914	DN914	Diabetic Nephropathy	0,04608
H7Glom.HGU133Plus2.DN916.DN916	DN916	Diabetic Nephropathy	2,7741
H7Glom.HGU133Plus2.DN932.DN932	DN932	Diabetic Nephropathy	0,71334
H7Glom.HGU133Plus2.DN941.DN941	DN941	Diabetic Nephropathy	0,07602
H7Glom.HGU133Plus2.DN947.DN947	DN947	Diabetic Nephropathy	1,59228

**Table S3. Pathways correlated with tubulointerstitial *SLC5A2* mRNA expression in diabetic nephropathy.**									
**Pathway**	**Entities found**	**Entities total**	**Entities ratio**	**Entities p**	**Entities FDR**	**Reactions found**	**Reactions total**	**Reations ratio**	**Species**
Metallothioneins bind metals	6	16	0.001	6.13E-7	5.39E-4	13	27	0.002	Homo sapiens
Response to metal ions	6	21	0.001	2.91E-6	1.28E-3	13	31	0.002	Homo sapiens
Metabolism	98	3,644	0.24	1.03E-5	3.02E-3	256	2,268	0.16	Homo sapiens
NR1H3 & NR1H2 regulate gene expression linked to	8	66	0.004	3.36E-5	7.36E-3	34	40		Homo sapiens
Metabolism of carbohydrates	19	459	0.03	8.27E-4	1.21E-1	28	241	0.017	Homo sapiens
Proline catabolism	3	14	0.001	2.23E-3	2.25E-1	3	4	0	Homo sapiens
Chylomicron assembly	3	14	0.001	2.23E-3	2.25E-1	1	5	0	Homo sapiens
Glyoxylate metabolism and glycine degradation	6	73	0.005	2.35E-3	2.25E-1	6	23	0.002	Homo sapiens
Biological oxidations	20	551	0.036	2.83E-3	2.25E-1	26	192	0.014	Homo sapiens
Plasma lipoprotein assembly, remodeling, and	7	102	0.007	2.85E-3	2.25E-1	14	86	0.006	Homo sapiens
Phase I - Functionalization of compounds	13	301	0.02	3.77E-3	2.38E-1	19	102	0.007	Homo sapiens
Plasma lipoprotein remodeling	5	56	0.004	3.81E-3	2.38E-1	7	31	0.002	Homo sapiens
Chylomicron remodeling	3	17	0.001	3.84E-3	2.38E-1	3	3	0	Homo sapiens
Keratan sulfate biosynthesis	4	36	0.002	4.44E-3	2.58E-1	5	9	0.001	Homo sapiens
Nuclear Receptor transcription pathway	6	86	0.006	5.19E-3	2.8E-1	2	2	0	Homo sapiens
HDL remodeling	3	24	0.002	9.86E-3	4.6E-1	1	13	0.001	Homo sapiens

Table S4. Pathways correlated with tubulointerstitial *C5* mRNA expression in diabetic nephropathy.									
**Pathway**	**Entities found**	**Entities total**	**Entities ratio**	**Entities p**	**Entities FDR**	**Reactions found**	**Reactions total**	**Reations ratio**	**Species**
Metabolism	276	3,644	0.24	1.11E-16	1.42E-13	538	2,268	0.16	Homo sapiens
Protein localization	34	170	0.011	9.43E-12	6.02E-9	47	53	0.004	Homo sapiens
Peroxisomal protein import	18	67	0.004	9.74E-9	4.14E-6	22	26	0.002	Homo sapiens
Metabolism of amino acids and derivatives	63	667	0.044	5.29E-7	1.63E-4	80	285	0.02	Homo sapiens
Aspirin ADME	17	80	0.005	6.4E-7	1.63E-4	10	26	0.002	Homo sapiens
Transport of small molecules	81	969	0.064	1.54E-6	3.26E-4	99	454	0.032	Homo sapiens
Mitochondrial Fatty Acid Beta-Oxidation	17	105	0.007	2.16E-5	3.94E-3	35	47	0.003	Homo sapiens
Metabolism of vitamins and cofactors	38	382	0.025	3.41E-5	4.81E-3	61	205	0.014	Homo sapiens
Drug ADME	27	239	0.016	6.14E-5	7.12E-3	28	121	0.009	Homo sapiens
PPARA activates gene expression	21	175	0.012	1.77E-4	1.73E-2	15	41	0.003	Homo sapiens
Regulation of lipid metabolism by PPARalpha	21	177	0.012	2.05E-4	1.87E-2	18	45	0.003	Homo sapiens
Iron uptake and transport	13	83	0.005	2.66E-4	2.26E-2	6	34	0.002	Homo sapiens
The citric acid (TCA) cycle and respiratory electron	25	238	0.016	3.33E-4	2.63E-2	38	67	0.005	Homo sapiens
Fatty acid metabolism	38	431	0.028	3.72E-4	2.63E-2	64	220	0.015	Homo sapiens
Biotin transport and metabolism	6	19	0.001	3.75E-4	2.63E-2	7	13	0.001	Homo sapiens
SLC-mediated transmembrane transport	37	418	0.028	4.12E-4	2.76E-2	38	194	0.014	Homo sapiens
Pyruvate metabolism and Citric Acid (TCA) cycle	14	100	0.007	4.76E-4	3,00E-02	17	36	0.003	Homo sapiens
Biological oxidations	45	551	0.036	5.63E-4	3.38E-2	70	192	0.014	Homo sapiens
Glucuronidation	10	58	0.004	6.43E-4	3.67E-2	8	11	0.001	Homo sapiens
Mitochondrial protein import	11	69	0.005	6.67E-4	3.67E-2	12	14	0.001	Homo sapiens
